# Draft Genome Sequence of *Streptomyces scabrisporus* NF3, an Endophyte Isolated from *Amphipterygium adstringens*

**DOI:** 10.1128/genomeA.00267-17

**Published:** 2017-04-27

**Authors:** Melissa Vazquez-Hernandez, Corina Diana Ceapa, Stefany Daniela Rodríguez-Luna, Romina Rodríguez-Sanoja, Sergio Sánchez

**Affiliations:** Instituto de Investigaciones Biomedicas, Universidad Nacional Autónoma de México, Ciudad de México, Mexico

## Abstract

We report the draft genome sequence of *Streptomyces scabrisporus* NF3, an endophyte isolated from *Amphipterygium adstringens* in Chiapas, Mexico. This strain produces a new modified linaridin peptide. The genome harbors at least 50 gene clusters for synthases of polyketide and nonribosomal peptides, suggesting a prospective production of various secondary metabolites.

## GENOME ANNOUNCEMENT

*Streptomyces scabrisporus* NF3, an endophytic actinomycete, isolated from the tree *Amphipterygium adstringens*, in Mexico (1), exhibits the potential to produce diverse bioactive compounds, for instance, the antibacterial hitachimycin (2) and the antitumoral alborixin (3). This species encompasses aerobic sporulating Gram-positive bacteria with a high G+C content (69 to 73%) (4). While other strains of the species have been isolated and characterized previously (3–6), only strain *S. scabrisporus* DSM 41855 was sequenced so far. The genome of this strain is composed of 199 contigs, making comparative analyses problematic.

The genome sequencing of *S. scabrisporus* NF3 was performed by the Yale Center for Genome Analysis (USA) using PacBio single-molecule real-time (SMRT) sequencing (two SMRT cells from a single library), after DNA extraction using a modified DNA isolation protocol (7). The sequencing of strain NF3 resulted in 2,035,773,977 bases in 130,565 reads, with an *N*_50_ mean read length of 28,885 bases. *De novo* assembly was performed using RS-HGAP2 in SMRT Portal. The draft genome sequence of 10,760,685 bp comprises eight scaffolds, the largest of them of 7,002,823 bp. The genome totals 10,492 coding sequences (CDSs), with 5,691 predicted genes and 4,729 hypothetical proteins, 60 tRNA genes, 12 rRNA operons, and a G+C content of 73%. The draft genome was annotated with Prokaryotic Dynamic Programming Genefinding Algorithm (PRODIGAL) and the Rapid Annotations using Subsystems Technology (RAST) (8, 9) server using the Pathosystems Resource Integration Center (PATRIC) (10).

Ribosomal 16S gene sequencing suggests a close similarity between *S. scabrisporus* strains NF3 and DSM 41855 (99.72%). Despite that, a proteome comparison of the two strains, performed in PATRIC (10), shows significant differences: 26% unique NF3 proteins (identity, <20%); 28% proteins between 20 and 80% identity, and only 46% of proteins identical between the two strains (>80%), partially due to the incompleteness of the type strain.

antiSMASH 3.0 (11) analysis of the *S. scabrisporus* NF3 genome revealed 50 secondary metabolites gene clusters: six type 1 polyketide synthase (PKS), two type II PKS, one type III PKS, 11 nonribosomal polyketide synthetase (NRPS), 10 PKS-NRPS hybrids, and 16 bacteriocins. Only two clusters shared complete identity with previously reported operons, and 17 clusters appear to be novel compared to *S. scabrisporus* DSM 41855 and showed reduced similarity to other prokaryote clusters. Interestingly, one cluster encodes a predicted linaridin, a rare linear ribosomally synthesized and posttranslationally modified peptide (RiPP) (12–14). The operon (21 kbp) contains seven predicted biosynthetic, two regulatory, and two transporter genes, of which 50% do not show similarity to known linaridin operon genes.

Potential host interaction molecules predicted using SignalP (15) include 657 signal peptide-containing genes. Remarkably, one WD-40 repeat protein, a eukaryotic protein that is common but rarely found in prokaryotes (16), was also identified.

This work indicates novel pathways of investigation for *S. scabrisporus* NF3, a notable antibiotic-producing strain, whose biologically active secondary metabolites and interaction with the host we plan to examine further.

### Accession number(s).

This whole-genome shotgun project has been deposited at DDBJ/ENA/GenBank under the accession no. MWQN00000000. The version described in this paper is version MWQN01000000.
