# Correction for Vazquez-Hernandez et al., “Draft Genome Sequence of *Streptomyces scabrisporus* NF3, an Endophyte Isolated from *Amphipterygium adstringens*”

**DOI:** 10.1128/genomeA.00583-17

**Published:** 2017-06-15

**Authors:** Melissa Vazquez-Hernandez, Corina Diana Ceapa, Stefany Daniela Rodríguez-Luna, Romina Rodríguez-Sanoja, Sergio Sánchez

**Affiliations:** Instituto de Investigaciones Biomedicas, Universidad Nacional Autónoma de México, Ciudad de México, Mexico

## AUTHOR CORRECTION

Volume 5, no. 17, e00267-17, 2017, https://doi.org/10.1128/genomeA.00267-17. Page 1: In line 2 of the abstract, “Chiapas, Mexico” should read “Morelos, Mexico.”

Page 1: In line 2 of the main text, the following should be cited in place of reference 1. “K. Rodríguez-Peña, M. Macías-Rubalcava, L. Rocha Zavaleta, R. Rodríguez-Sanoja and S. Sanchez, unpublished data.”

Page 2: Reference 1 should read “Reference deleted.”

